# Sexual and reproductive health and gender-based violence among female migrants in Morocco: a cross sectional survey

**DOI:** 10.1186/s12905-023-02307-1

**Published:** 2023-04-11

**Authors:** Laila Acharai, Mohamed Khalis, Oumnia Bouaddi, Ghida Krisht, Sanae Elomrani, Abdelhakim Yahyane, Bouchra Assarag

**Affiliations:** 1 International School of Public Health, Mohammed VI University of Health Sciences, Casablanca, Morocco. Boulevard Mohammed Taïeb Naciri, Commune Hay Hassani Casablanca, Casablanca, 82403 Morocco; 2 Higher Institute of Nursing Professions and Technical Health, Rabat, Morocco; 3 Mohammed VI Center of Research and Innovation, Rabat, Morocco; 4 Knowledge for Health Policies Center, Casablanca, Morocco; 5 American University of Beirut, Beirut, Lebanon; 6 National School of Public Health, Rabat, Morocco; 7 Direction of Population, Ministry of Health and Social Protection, Rabat, Morocco

**Keywords:** Migrants, Sexual violence, Gender-based violence, Sexual and reproductive health, Morocco

## Abstract

**Background:**

Over the past decade, Morocco has increasingly become the chosen destination for a growing number of migrants from neighbouring countries and especially from Sub-Saharan Africa. The aim of this study is to describe the sexual and reproductive health (SRH), as well as sexual and gender-based violence (SGBV) among female migrants in Morocco.

**Methods:**

This is a descriptive cross-sectional study conducted between July and December 2021. Female migrants were recruited from one university maternity hospital and two primary healthcare centres in Rabat. Data were collected using a structured face-to-face questionnaire, which included information about sociodemographic characteristics, SRH, history of SGBV and its impact, as well as the utilization of preventive and supportive SGBV services.

**Results:**

A total of 151 participants were included in this study. The majority of participants (60.9%) were aged 18 to 34 years old and 83.3% were single. Many participants (62.1%) did not use contraceptives. More than half (56%) of the participants who were pregnant at the time of the study were receiving pre-natal care. About 29.9% of interviewed participants reported experiencing female genital mutilation, and a significant majority (87.4%) experienced SGBV at least once during their lifetimes, while 76.2% experienced SGBV during migration. The most commonly reported form of violence was verbal abuse (75.8%). Among the victims of SGBV, a minority have visited a health facility (7%) or filed a complaint (9%) in the aftermath of violence.

**Conclusion:**

Overall, our findings showed low contraception coverage, moderate access to prenatal care, high prevalence of SGBV, and low utilization of preventive and supportive SGBV services among migrant women in Morocco. Further studies are needed to understand the contextual barriers to access, and utilization of SRH care and additional efforts should be undertaken to strengthen SGBV prevention and support systems.

**Supplementary Information:**

The online version contains supplementary material available at 10.1186/s12905-023-02307-1.

## Introduction

Migration has become a pervasive global phenomenon over the past decade [1]. According to the International Organization of Migrants (IOM), there were 281 million migrants in the world in 2020, which make up 3.6 per cent of the global population [1]. Migrants often come from countries where they experienced political instability, economic austerity, and structural violence [2]. Concurrently, migrants experience additional hardships during their migration pathway or in the host country, such as marginalization, discrimination, violence in all forms, abuse and exploitation [2]. Consequently, the migrant population often has poor social, economic, psychological and health outcomes, making migration a troubling global human rights and public health issue [3]. In fact, a robust body of evidence shows that migrants are disproportionately affected by mental health conditions, chronic diseases, antimicrobial resistance, occupational hazards, and oral diseases compared to the host population [4–7]. Additionally, migrants have been shown to have lower immunization rates compared to the host populations [8]. Furthermore, migrants, especially women and girls, – often experience poor sexual and reproductive health (SRH) outcomes such as low skilled-birth attendance [9], unintended pregnancies and abortion [10], inadequate pre-natal care [11] and low contraception coverage [12].

SGBV is a global phenomenon which affects an estimated 30% of ever-married or partnered women aged between 15 and 49 years old worldwide [13]. In the Eastern Mediterranean Region, 31% of ever partnered women experienced physical and/or sexual intimate partner violence during the course of their lives [14]. SGBV results in poor SRH and mental health outcomes among the victims [15]. In fact, according to the World Health Organization (WHO), women who have been physically or sexually abused are more likely to experience unintended pregnancies, gynecological morbidities, miscarriages, unsafe abortions, pregnancy complications and sexually-transmitted infections including HIV [15]. Consequently, the 70th World Health Assembly resolution stressed the importance of a multisectoral national response to interpersonal violence, particularly against women, girls and children, and endorsed a Global Plan of Action to strengthen the role of the health system in spearheading this endeavour [16]. Migrant women and girls are often more vulnerable to SGBV given the harsh migration journey and the difficult socio-economic conditions they experience in the host countries [2].

The number of migrants has been growing over the past decade in Morocco, the chosen country of destination for migrants from neighbouring countries, especially Sub-Saharan Africa [17]. The International Organization for Migration estimates that Morocco was home to 102 thousand migrants as of 2021, of which half were female [1]. Based on a national survey of 3000 migrants and refugees in Morocco, conducted by the High Commission of Planning (HCP) in 2021, the Democratic Republic of the Congo (DRC) (19.1%), Ivory coast (16.7%), Senegal (15.9%) and Guinea (13.2%) were the main countries of origin of migrants in Morocco [17]. Two consecutive mass regularization campaigns in 2014 and 2017 resulted in the regularization of almost 50,000 migrants including unaccompanied minors and more than 10,000 women [17]. Several efforts geared towards improving the living conditions of migrants and their access to public services have been undertaken by the Moroccan government, such as the adoption of the Global Compact for Safe, Orderly and Regular Migration in 2018[18] and the implementation of the National Strategy for Immigration and Asylum [19]. Moreover, the Moroccan Ministry of Health and Social Protection has been engaged, in collaboration with civil society and non-governmental organizations (CSOs/NGOs), in a participatory process aimed at improving access to healthcare services by the migrant population. In 2017, a circular was signed by the Minister Delegate to the Minister of the Interior, the Minister in charge of Moroccans residing abroad, the Minister in charge of Economy and Finance, and the Minister of Health, that provides documented migrants and refugees with access to multiple healthcare services under the Moroccan Health Insurance Scheme for the Economically Deprived (RAMED) [20]. With support from the IOM, the Ministry of Health also developed the National Strategic Plan for Health and Immigration (PSNSI) which covers the period 2021–2025 with the aim of improving access to health among migrants through an integrated and comprehensive approach [20]. The PSNSI identified the following areas of focus: strengthening monitoring, evaluation and research; building the capacity of different actors involved in promoting the health of migrants; the promotion of health and preventive care; provision of adequate care and good governance [20]. Regular migrants in Morocco have the right to access free of charge emergency care and could benefit from healthcare services in a wide network of primary healthcare facilities across the country. In addition, migrants are included in national vertical programs put in place by the Ministry of Health such as Family Planning, Maternal and Child Health and Reproductive health care programs [21]. The 2018, the Ministry of Health with help from NGOs, organized a national two-month TB screening campaign with Morocco’s migration organization partners. During this campaign, 5,553 migrants were tested for HIV, and 12,013 migrants received information on HIV prevention [22]. In addition, a winter school on Sexual and Reproductive Health Rights (SRHR) was organized by the Ministry of Health in collaboration with IOM in order to improve knowledge of SRH among migrants in Morocco. Moreover, several interventions aimed at improving the cultural competency of healthcare professionals have been implemented and a toolkit was developed to increase healthcare professionals’ sensitivity to migrant health issues [23]. Nevertheless, the utilization of healthcare services by migrants in Morocco remains insufficient due to several contextual and structural barriers such as language and cultural barriers, the lack of culturally-competent healthcare professionals and the lack of information on healthcare services [21]. In Morocco, data on the SRH outcomes of migrant women remains scarce, and very few studies have explored migrant women’s exposure to SGBV. In 2021, around 11.7% of migrant women in Morocco experienced physical or psychological violence, 17.7% have been victims of forced sexual intercourse or sexual harassment and 4.3% were pregnant or gave birth during their travel [17]. NGOs and CSOs in Morocco undertake tremendous efforts to provide psychological support to victims of SGBV including migrants. However, the utilization of SGBV prevention and support services psychosocial by migrants has not been the subject of previous research. The aim of this study is to describe the SRH as well as the experiences of SGBV among female migrant population in Morocco. The findings of this study will be useful to identify areas for improvement with regards to access and utilization of SRH and SGBV prevention and support services by the migrant population in Morocco.

## Materials and methods

### Study design

This is a descriptive cross-sectional study conducted between July and December 2021 in Rabat, Morocco.

### Study population

Female migrants were recruited from one maternity university hospital (Les Orangers Maternity Hospital) and two primary healthcare centres in Rabat. The healthcare facilities in Rabat were selected due to the high influx of migrants. The inclusion criteria were being a migrant woman, regardless of legal status, aged 18 years old and over and having benefited from a medical consultation regarding SRH in one of the selected healthcare facilities. Participants were recruited through convenient sampling. The sample size was calculated using the OpenEpi, version 3 software (www.OpenEpi.com).

$$\eqalign{&{\rm{n}} = \cr &[ {\rm{DEFF}}^*{\rm{Np}}( {1 - {\rm{p}}} ) ]/[({{\rm{d}}^2}/{{\rm{Z}}^2}_{\rm{1 - \alpha /2^*}}( {{\rm{N}} - 1} ) + {\rm{p}}^*( {1 - {\rm{p}}})]}$$

N is Population size (for finite population correction factor or fpc) = 51,200; p is the hypothesized (%) frequency of outcome factor in the population (10% +/−5; Confidence limits as % of 100) (absolute +/−%)(d) = 5%; DEFF (design effect = 1); Z is a constant = 1.96 for 95% Confidence interval. Based on the above parameters the minimum required sample size (n) was 138 participants.

### Data collection

Data were collected through face-to-face interviews by three health care professionals, working at the selected healthcare facilities. The three interviewers were trained by the research team prior to the study. Structured questionnaires were administered before the consultations. Participants were given a unique identifier in order to avoid duplication. Once a participant was interviewed, the trained interviewers were instructed to cross their identifier off the list.

The questionnaire was developed based on existing literature and previous experience[24–26] (Additional File). The questionnaire comprised of three sections. The first section included general information (age, marital status, country of origin, duration of stay in Morocco, language, level of education, occupation, reasons for leaving country of origin, transit in Morocco, migration status, monthly income, social security, place of residence, household size). The second section included information about the SRH of the study participants (history of pregnancy, experience giving birth in a health facility, current use of contraceptives, history of unintended pregnancies, current pregnancy status, receiving pre-natal care, current diagnosis with obstetric morbidities). The third section included questions about SGBV (history of female genital mutilation, history of SGBV during lifetime and during migration, relationship with the aggressors, forms of SGBV, approaches used to commit SGBV). The fourth section included questions about the impact of SGBV (avoiding going out alone, change of residence, change in daily habits, experiencing psychological repercussions, experiencing repercussions on sexual health), and the utilization of prevention and support services (consulting a lawyer, reaching out to a CSO/NGO, visiting a health facility, filing a complaint). The questionnaire was administered in French for the majority of participants. One participant spoke English, therefore the questionnaire was translated into English and validated by the research team. Another participant spoke a foreign dialect but could understand French.

## Operational definitions

SGBV was defined as “any harmful acts directed against a person because of his or her sex or gender. It includes sexual, physical, mental and economic harm inflicted in public or in private. It also includes threats of violence, coercion and manipulation [27].

Physical violence was defined as “the intentional use of physical force with the potential to cause death, disability, injury or harm” [28].

Sexual violence was defined “any sexual act, attempt to obtain a sexual act, or other act directed against a person’s sexuality using coercion, by any person regardless of their relationship to the victim, in any setting. It includes rape, defined as the physically forced or otherwise coerced penetration of the vulva or anus with a penis, other body part or object, attempted rape, unwanted sexual touching and other non-contact forms“[29].

Verbal abuse was defined “an act that undermines a person’s sense of self-worth through constant criticism; belittling one’s abilities; name-calling or other forms of insults”[30].

Blackmail was defined as “the act of threatening to harm someone or someone’s reputation unless the person does as you say, or a payment made to someone who has threatened to harm another person or another person’s reputation if they fail to pay them” [31]. Intimidation was defined as “the action of frightening or threatening someone, usually in order to persuade them to do something that you want them to do”[32].

Sexual dysfunction was defined as “any difficulty moving through the stages of sexual desire, arousal, and orgasm, as well as subjective satisfaction with the frequency and outcome of individual and partnered sexual behavior”[33]. Obstetric morbidities were defined as “any condition that is attributed to or aggravated by pregnancy and childbirth which has a negative impact on the woman’s wellbeing and/or functioning” [34].

### Ethical considerations

This study was approved by the Ethics Committee. All study participants were given an information sheet about the aim and objectives of the study. Participants were also made aware of their right to withdraw from the study at any moment. Complete anonymity was maintained to guarantee confidentiality of data and protect the identity of the study participants.

### Statistical analysis

Statistical analysis was conducted using SPSS software (version 21). Quantitative variables were presented in means and standard deviations (means ± standard deviation) and qualitative variables were presented in numbers and percentages (%).

## Results

### Socio-demographic characteristics of the study population

Of 151 participants included in this study, many participants (60.9%) were between the ages of 18 and 34 years old and 83.3% were single. The countries of origin included the Ivory coast (28.5%), the Democratic Republic of Congo (DRC) (17.9%), Guinea Conakry (24.5%) and Cameroun (20.5%). Almost all participants spoke French (98.7%) and the majority either received primary or secondary education (51%) or higher education or vocational training (29.1%). However, approximately 19.9% of respondents had no prior education. The majority of migrants (72.2%) in this study have been in Morocco for less than four years. Approximately 82% of participants declared having no source of income, and the majority (96.4%) did not have social security. A significant share of respondents (80.1%) considered Morocco a country of transit (Table 1).

**Table 1:** Socio-demographic characteristics of the study population (n = 151)

Variables	Distribution n (%)
Age (years)	
18 to 34	92 (60.9)
35 to 54	56 (37.1)
≥ 55	3 (2.0)
Marital status	
Single	125 (83.3)
Married	16 (10.7)
Divorced	6 (4.0)
Widowed	3 (2.0)
Missing	1
Country of origin	
Ivory Coast	43 (28.5)
Democratic Republic of Congo	27 (17.9)
Guinea Conakry	37 (24.5)
Cameroun	13 (20.5)
Other	31 (20.5)
Missing	33
Duration of stay in Morocco (years)	
< 4	109 (72.2)
4 to 8	24 (15.9)
≥ 8	18 (11.9)
Language	
French	148 (98.7)
Other	2 (1.3)
Missing	1
Level of education	
No prior education	30 (19.9)
Primary/secondary education	77 (51.0)
University/vocational training	44 (29.1)
Occupation	
Salaried employee	25 (18.9)
Independent worker	17 (12.9)
Other	90 (68.2)
Missing	19
Reasons for leaving country of origin	
Escape political conflict	11 (7.3)
Improve quality of life	119 (78.8)
Reunite with family	13 (8.6)
Pursue studies	8 (5.3)
Transit in Morocco	
Transit country	113 (80.1)
Destination country	28 (19.9)
Missing	10
Migration status	
Unauthorized migrant	46 (30.5)
Refugee or asylum seeker	73 (48.3)
Authorized migrant	32 (21.2)
Monthly income (USD^a^)	
No income	82 (82.0)
< 95	4 (4.0)
≥ 95	14 (14.0)
Missing	51
Social security	
Non-beneficiary	133 (96.4)
Beneficiary	5 (3.6)
Missing	13
Place of residence	
No fixed place of residence	1 (0.7)
Shanty town	1 (0.7)
Room/Studio/Apartment	149 (98)
Household size	3.80 ± 1.41

^a^USD: United States Dollars.

### Sexual and reproductive health of migrant women in Morocco

In this study, approximately 76.7% of participants had history of pregnancy, of which 67.3% previously gave birth in a health facility. Among respondents, the majority (62.1%) did not use any contraceptive method and approximately 11.7% have previously experienced an unintended pregnancy. About 16.6% of women were pregnant at the time of the study among which 56% declared receiving pre-natal care. Finally, approximately 12.1% of women with a history of pregnancy had an obstetric morbidity at the time of the study (Table 2).

**Table 2:** Sexual and reproductive health of migrant women (n = 151)

Variables	Yes n (%)	No n (%)
History of pregnancy (n = 150)	115 (76.7)	35 (23.3)
Experience giving birth in a health facility^a^	66 (67.3)	61 (32.7)
Current use of contraceptives (n = 145)	55 (37.9)	90 (62.1)
History of unintended pregnancies (n = 145)	17 (11.7)	128 (88.3)
Currently pregnant	25 (16.6)	126 (83.4)
Currently receiving pre-natal care^b^	14 (56.0)	11 (44.0)
Currently diagnosed with an obstetric morbidity^c^ (n = 115)	13(12.13)	94(87.9)

^a^ Among parous women.

^b^ Among women who are currently pregnant. ^c^ Among women with history of pregnancy.

### Sexual and gender-based violence against migrant women in Morocco

In this study, almost 30% of participants have experienced female genital mutilation. The majority of participants (87.4%) have experienced SGBV during their lifetime and 68.7% have been victims of SGBV more than once. Additionally, more than two-thirds of participants (76.2%) have experienced SGBV during migration. Among victims of SGBV during migration, 21.2% have been aggressed by a thief or a delinquent. A large share of SGBV victims (75.8%) have experienced verbal abuse and 21.2% have experienced physical violence. When asked about the approaches used by the aggressor, over half of the study participants (58.6%) declared having been threatened with arms and/or physical force and 24.2% have been subject to intimidation (Table 3).

**Table 3:** History and forms of SGBV among study participants (n = 151)

Variables		Yes n (%)	No n (%)
History of female genital mutilation (n = 147)		44 (29.9)	103 (70.1)
History of SGBV during lifetime		132 (87.4)	19 (12.6)
Multiple exposure to SGBV during lifetime		90 (68.7)	41 (31.3)
History of SGBV during migration ^a^		99 (76.2)	31 (23.8)
Relationship with the aggressor ^b^			
	Intimate partner	3 (3.0)	96 (97.0)
	Family member	2 (2.0)	97 (98.0)
	Delinquent or thief	21 (21.2)	78 (78.8)
	Other	73 (73.8)	26 (26.2)
Forms of SGBV ^b^			
	Verbal abuse	75 (75.8)	24 (24.2)
	Physical violence (e.g., for theft)	21 (21.2)	78 (78.8)
	Sexual abuse	2 (2.0)	97 (98.0)
Approaches used by the aggressor to commit violence ^b^			
	Intimidation	24 (24.2)	75 (75.8)
	Blackmail	2 (2.0)	97 (98.0)
	Threatening to use arms and/or physical force	58 (58.6)	41 (41.4)

^a^ Among victims of SGBV.

^b^ Among victims of SGBV during migration (n = 99).

### Impact of SGBV and utilization of preventive and supportive SGBV services by migrant victims

Among victims of SGBV during migration, only a minority have consulted a lawyer (1%), or have or reached out to a CSO/NGO (2%), or visited a health facility (7%) or filed a complaint (9%). Among victims who filed a complaint, only 9.1% declared having received follow-up on their complaint. Among victims of SGBV during migration, the majority (87.9%) avoid going out alone, 14.1% changed their place of residence, and 73.7% changed their daily habits. When asked about psychological repercussions, only a minority of victims (10.1%) declared having experienced psychological repercussions that required professional help in the aftermath of the violent act. Finally, only a minority of victims (2%) reported having experienced disruptions in their sexuality in the aftermath of the violent act (Table 4).

**Table 4:** The impact of SGBV and utilization of preventive and supportive SGBV services by migrants who experienced SGBV during migration (n = 99)

Variables	Yes n (%)	No n (%)
Talking to someone	89 (89.9)	10 (10.1)
Consulting a lawyer	1 (1.0)	98 (99.0)
Reaching out to a CSO/NGO^a^	2 (2.0)	97 (98.0)
Visiting a health facility	7 (7.0)	92 (93.0)
Filing a complaint	9 (9.0)	90 (91.0)
Follow-up on the complaint^b^	1 (9.1)	10 (90.9)
Receiving financial aid	1 (1.0)	98 (99.0)
Avoiding going out alone	87 (87.9)	12 (12.1)
Change of residence	14 (14.1)	85 (85.9)
Change in daily habits	73 (73.7)	26 (26.3)
Suffering psychological repercussions which required professional help	10 (10.1)	89 (89.9)
Sexual dysfunction	2 (2.0)	97 (98.0)

^a^ CSO/NGO: Civil Society/ Non-Governmental Organizations.

^b^ Among victims who filed a complaint.

## Discussion

This study described the SRH outcomes of migrant women in Morocco as well as the prevalence of SGBV and utilization of support services. The main findings were that many migrant women (62.1%) in this study did not use any contraceptives. However, the majority of women who were pregnant at the time of the study were receiving pre-natal care. In addition, a high share of participants in this study reported being a victim of SGBV, especially verbal, physical and psychological violence, and have expressed the impact of the violence on their daily lives. Finally, the utilization of legal, healthcare and social support services was low among victims of SGBV.

In this study, over half of migrant women who were pregnant at the time of study declared receiving pre-natal care. However, over two thirds of migrant women were not using any contraceptive method. In the general population, according to recent data released by the United Nations Population Fund (UNFPA), the prevalence rate of contraception use is 43% among all women and 71% among married and in union women as of 2022,[35]. Furthermore, 87% of births were attended by skilled health personnel between 2004 and 2020 [35]. Family planning represents a major challenge to population health in Morocco due to its social, economic and health implications. Major strides have been made in contraception coverage, reducing maternal and child mortality, preventing unintended pregnancies and the provision of pre-natal care [36]. Local NGOs have launched several initiatives to promote the SRH of migrants in Morocco. In 2018, community-based health services launched by the Moroccan Association of Family Planning have raised awareness about family planning reaching 16,400 men and women, including migrants, refugees and asylum seekers [37]. In 2017, 329 women have benefited from pregnancy surveillance through the National Program for Pregnancy and Childbirth Surveillance, in three Moroccan regions [38]. Additionally, about 224 and 534 women benefited from family planning services and HIV-AIDS follow-up respectively in the same regions, and 57 migrant women were admitted to a maternity hospital [38]. Despite the abovementioned efforts to improve access to care, there remains an underutilization of healthcare services by the migrant population which spurs questions around the local barriers that prevent migrants from accessing services offered by Ministry of Health and its NGO and civil society partners. Migrant women and girls are subject to the interruption of the continuum of care during their migration or in the host country and are therefore at high risk of pregnancy complications and morbidities. Consequently, there would seem to be a definite need for further large-scale studies in order to identify institutional and structural barriers that impede access to care by the migrant population in Morocco. These barriers would be an appropriate target for interventions aimed at improving health outcomes of migrant women and facilitating their social and professional integration.

In this study, that most participants (76.2%) declared having been a victim of SGBV during migration. This number is lower compared to another study among sub-Saharan migrants in Morocco and along its borders (89.6%) [39]. Lower numbers were reported among migrants in Italy 46.5% [26], among female refugees and asylum seekers in the DRC, Somalia and Uganda (52.1%) [40], among immigrants in the United States (4.1%)[41] and among Moroccan migrants in Spain (12%) [7]. Verbal abuse followed by physical violence were the most common forms of SGBV in this study which match the results of a study among immigrants and refugees in Italy [26]. Psychological violence such as threats to use arms and/or physical force was also common among survivors of SGBV in this study. It is often difficult to determine the extent of violence among migrant populations; however, data from organizations such as Médecins Sans Frontières (MSF) showed that in 2012, care has been provided to approximately 700 survivors of sexual violence in Morocco, among which 94% were female [42]. In 7% of cases, there were more than two aggressors and the majority of victims were from the DRC (32%) and Nigeria (30%) [42]. Additionally, one qualitative study using a community-based research approach explored violence among sub-Saharan migrants in Morocco [39]. Results showed that much of the violence was of a sexual nature which is differs greatly from the findings in this study, where only 2% of SGBV victims reported a sexual form of abuse [39]. The forms of SGBV experienced by migrants often vary during migration phases and may lead to migrants experiencing several forms of SGBV and by different aggressors. Further understanding of how time and space affect the experience of SGBV should be the target of future research and would guide the design of appropriate interventions tailored to migration stages and forms of SGBV [43].

In this study only a minority of survivors of SGBV reported the incident. Underreporting largely affects the estimations of the magnitude of SGBV. Some studies exploring the determinants of reporting of SGBV have shown that reporting was less likely to occur among women with no education or with a low education level which make up a small but non-negligible percentage of migrant women in this study. However, some contradicting findings were reported by other studies where reporting of SGBV was lower among women with higher than primary school education. This was explained by the need of highly educated survivors to maintain their social standing in their communities [44]. Other factors such as shame, guilt and stigma particularly in the case of sexual violence, doubts about confidentiality and about being believed, act as barriers to the reporting of SGBV [45]. Furthermore, studies among survivors of SGBV showed that women who relied on financial support from their partners were also less likely to report SGBV. Many NGO-led initiatives in Morocco are geared towards capacity building and improving professional integration of migrants within their host communities [46]. These efforts need to be strengthened in order to promote autonomy among migrant women and empower them to report SGBV, as well as to seek and utilize support services, particularly in the case of intimate partner violence.

The precarious conditions under which migrants, especially undocumented migrants live, and their exposure to violence and abuse increases their vulnerability as well as their healthcare and psychological needs. While major progress has been made in Morocco with regards to recognizing the rights of migrants, and more specifically their right to adequate healthcare [20], more efforts should go towards understanding and addressing the contextual determinants of vulnerability [2]. In this study, only a minority of SGBV victims used one or more form of SGBV support services. Potential causes of the underutilization of these services are lack of adequate information systems and communication channels, previous unsatisfying experiences, stigma and discrimination and fear of deportation in the case of lack of legal documentation [47]. Further research is needed to investigate these barriers and identify facilitators that strengthen SGBV support systems. Nevertheless, the need to provide information about available SGBV support services to migrant women Morocco cannot be overemphasized. Lastly, improving coordination mechanisms and referral pathways between NGOs offering these services and healthcare facilities in Morocco, is critical in order to mitigate the negative SRH outcomes in victims of SGBV.

This is the first descriptive study to describe the SRH status of migrant women in Morocco and the first study to explore the impact of SGBV and the utilization of preventive and supportive SGBV services by the victims. Our study has some limitations. First, the study was only performed at three centres; therefore, the results should be generalized with caution to the total migrant population in Morocco. Second, the findings were based on self-reported responses which may have been subject to recollection bias. Third, the sensitive nature of the topic may lead to underreporting especially pertaining to sexual violence, which could potentially lead to the underestimation of the real prevalence of certain forms of SGBV. Fourth, this study was conducted in healthcare settings which may have affected the prevalence estimates.

## Conclusion

Overall, our findings show a low contraception coverage, moderate access to prenatal care, high prevalence of SGBV, and low utilization of preventive and supportive SGBV services by migrant women in Morocco. Despite the efforts undertaken by the Moroccan government and the Ministry of Health, the SRH needs of the migrant population are yet to be realized. The complexity of the migration phenomenon and its spill-over consequences on health and social well-being call for comprehensive public policies that address the fundamental determinants of the vulnerability experienced by this population. Because of the intimate link between SGBV and SRH outcomes, it is also imperative that SGBV is included as a strategic area of focus and is integrated in the national 2021–2030 SRH Strategy in Morocco.

## Electronic supplementary material

Below is the link to the electronic supplementary material.

**Additional File**: Sexual and reproductive health and gender-based violence among female migrants in Morocco: a cross sectional survey

## Data Availability

The datasets used and/or analyzed during the current study are available from the corresponding author upon reasonable request.

## Abbreviations

SRH: Sexual and reproductive health
SGBV: Sexual and gender-based violence
IOM: International Organization for Migration
CSO: Civil Society Organization
NGO: Non-Governmental Organization
HCP: High Commission of Planning
WHO: World Health Organization
UNFPA: United Nations Population Fund
