# Supplementary material for: Sexual and reproductive health and gender-based violence among female migrants in Morocco: a cross sectional survey
Source: BMC Womens Health. 2023 Apr 11;23:174. doi: 10.1186/s12905-023-02307-1 (PMC10091612; doi:10.1186/s12905-023-02307-1)
Supplement: Supplementary file 1 — Additional File: Sexual and reproductive health and gender-based violence among female migrants in Morocco: a cross sectional survey [file 12905_2023_2307_MOESM1_ESM.docx]

**Sexual and reproductive health and gender-based violence among female migrants in Morocco: a cross sectional survey**

This questionnaire is part of the study entitled " Sexual and reproductive health and gender-based violence among female migrants in Morocco: a cross sectional survey ". It is intended for migrant women residing in Morocco. Our research aims to contribute to a better understanding of the occurrence of sexual and gender-based violence against migrant women and girls in Morocco in order to improve the quality of available services.

Your opinion is very valuable to us, as it will help us improve the living conditions of migrants. Please answer the questions below by ticking the boxes. We assure you that the information to be collected will be anonymous and confidential.

**GENERAL INFORMATIONS:**

| **Participant ID: ..............**  **Province: ...................................... Name of healthcare facility........ .................................................**  **Date: ___/___/___** |
| --- |

**Part I: General information**

1. **How old are you?**

- From 18 to 34 years old
- From 35 to 54 years old
- Over 55

1. **Marital status:**

- Single
- Married
- Divorced
- Widowed

1. **Do you have children:**

- No
- Yes

1. **If you have children, how many do you have**

**……………………………………………………………………………………………………………………………**

1. **Country of origin:** …………………………………
2. **Length of stay in Morocco**

- Less 4 years old
- Less than 8 years
- More than 8 years

1. **: Language**

- French
- English
- Arabic
- Other

If other, which one? .............................................. .........

1. **Educational level:**

- None
- Primary
- Secondary
- University
- Vocational training

1. **Professional status**

- Salaried employee
- Independent worker
- Other

1. **: Reason for leaving**

- Escape political conflict
- Improve quality of life
- Join your family
- Pursue studies

1. **: When you came to Morocco, were you planning to**

- Stay here
- Just passing through

1. **: Legal status**

- Regular
- Refugee
- Asking for asylum
- Without papers

1. **Do you have health insurance?**

- Yes
- No

1. **What is your monthly income**

- No income
- Less than 1000 DH
- More than 1000 DH

1. **What is your place of residence?**

- No fixed place of residence
- Room
- Studio
- Apartment
- Shanty town

1. **How many people are currently residing with you?**

**………………………………………………………………………………..……………………………………**

**Part II: Sexual and reproductive health**

1. **Have you been pregnant before?**

- Yes
- No

1. **Are you currently pregnant?**

- Yes
- No

1. **Do you benefit from follow-up of your pregnancy?**

- Yes
- No

1. **Have you ever given birth in a health facility?**

- Yes
- No

1. **Are you using a contraceptive method?**

- Yes
- No

1. **Have you ever become pregnant unintentionally (unwanted pregnancy)?**

- Yes
- No

1. **Do you suffer from any obstetric morbidities? (a health condition linked to or aggravated by pregnancy)**

- Yes
- No

**Part III: Exposure to sexual and gender-based violence**

1. **Have you undergone genital mutilation? (genital cutting)**

- Yes
- No

1. **Have you ever experienced violence in your lifetime?**

- Yes
- No

1. **Have you ever experienced violence more than once in your lifetime?**

- Yes
- No

1. **Have you ever experienced violence during migration**

- Yes
- No

**If yes, what type:**

- Verbal violence (e.g. insults)
- Physical violence (e.g., theft)
- Sexual abuse

If other, please specify ………………………

1. **How were you forced into these acts?**

- Threat of arm or physical force
- Intimidation
- Blackmail
- Threats to others

1. **Who was the aggressor?**

- Husband/Partner
- Delinquent or thief
- Family member
- Other

If other, please specify ………………………

**Part IV: Impact and utilization of support services**

1. **Following this event, did you talk to someone?**

- Yes
- No

1. **Following this event, did you avoid going out alone?**

- Yes
- No

1. **Following this event, did you or another person contact a support association, call a support number?**

- Yes
- No

1. **Following this event, did you receive financial aid?**

- Yes
- No

1. **Following this event, did you move out of your place of residence?**

- Yes
- No

1. **Following this event, did you change your daily habits? (ex: travelling, going out)**

- Yes
- No

1. **Following this event, did you or another person consult a lawyer?**

- Yes
- No

1. **Following this event, did you or another person consult a health facility?**

- Yes,
- No

1. **Following this fact, did you file a complaint?**

- Yes
- No

1. **If yes, was any legal action taken following the complaint?**

- Yes
- No

1. **Following this event, did you experience psychological issues which required professional help?**

- Yes
- No

1. **Following this event, did you experience sexual dysfunction (ex: disruption to the ability to feel pleasure, orgasm, or arousal)**

- Yes
- No

**Thank you for your sincere cooperation**
